# GWAS for urinary sodium and potassium excretion highlights pathways shared with cardiovascular traits

**DOI:** 10.1038/s41467-019-11451-y

**Published:** 2019-08-13

**Authors:** Raha Pazoki, Evangelos Evangelou, David Mosen-Ansorena, Rui Climaco Pinto, Ibrahim Karaman, Paul Blakeley, Dipender Gill, Verena Zuber, Paul Elliott, Ioanna Tzoulaki, Abbas Dehghan

**Affiliations:** 1 MRC-PHE Centre for Environment and Health, Department of Epidemiology and Biostatistics, School of Public Health, St Mary’s campus, Norfolk Place, London, W2 1PG UK; 2 Department of Hygiene and Epidemiology, University of Ioannina Medical School, 45110 Ioannina, Greece; 3 Dementia Research Institute at Imperial College London, London, W2 1PG UK; 4 NIHR Imperial Biomedical Research Centre, ITMAT Data Science Group, Imperial College London, London, W2 1PG UK; 5 Department of Stroke Medicine, Imperial College London, London, W2 1PG UK; 6 Imperial College NIHR Biomedical Research Centre, London, W2 1NY UK; 7 Health Data Research UK-London, London, NW1 2BE UK

**Keywords:** Genome-wide association studies, Cardiovascular genetics, Kidney, Risk factors

## Abstract

Urinary sodium and potassium excretion are associated with blood pressure (BP) and cardiovascular disease (CVD). The exact biological link between these traits is yet to be elucidated. Here, we identify 50 loci for sodium and 13 for potassium excretion in a large-scale genome-wide association study (GWAS) on urinary sodium and potassium excretion using data from 446,237 individuals of European descent from the UK Biobank study. We extensively interrogate the results using multiple analyses such as Mendelian randomization, functional assessment, co localization, genetic risk score, and pathway analyses. We identify a shared genetic component between urinary sodium and potassium expression and cardiovascular traits. Ingenuity pathway analysis shows that urinary sodium and potassium excretion loci are over-represented in behavioural response to stimuli. Our study highlights pathways that are shared between urinary sodium and potassium excretion and cardiovascular traits.

## Abstract (web summary)

Levels of sodium and potassium in urine are associated with cardiovascular traits. Here, Pazoki *et al*. perform genome-wide association studies for urinary sodium and potassium secretion and identify 50 and 13 novel loci, respectively, some of which show a potential causal relationship with blood pressure based on MR analysis.

## Introduction

Cardiovascular disease (CVD) leads to 17.5 million annual deaths worldwide^1^. Sodium consumption is a risk factor of CVD and is estimated to have caused 1.65 million CVD deaths in 2010^2^. Urinary sodium and potassium excretion have been associated with blood pressure (BP) and cardiovascular events^3–6^. At the same time, epidemiological studies, animal models, and clinical trials support a strong link between sodium intake^7^, and BP;^2–4,6,8–10^ the leading modifiable cause of morbidity and mortality from CVD worldwide. Biological mechanisms that link these traits together are not exactly clear. To understand the genetic and physiological pathways underlying these electrolytes and their link to BP and cardiovascular events, we undertake a genome-wide association study (GWAS) on urinary sodium and potassium excretion among 446,237 European individuals in UK Biobank (UKB)^11–13^. Here, we identify 50 sodium and 13 potassium novel loci at stringent threshold of *P* < 1 × 10^−8^. Most of these loci had previously been found to be associated with lipid levels, anthropometric and lifestyle traits such as dietary intake, smoking-related behavior, and alcohol consumption at GWAS significance level (*P* < 5 × 10^−8^). In pathway analyses, sodium and potassium excretion loci are over-represented in biological functions involving behavioral response to stimuli, thermoregulation, and weight loss. A subset of loci involved in behavioral response to stimuli support a link to BP and coronary artery disease based on Mendelian randomization (MR) analysis.

## Results and discussion

### Main findings

**Fig. 1:**
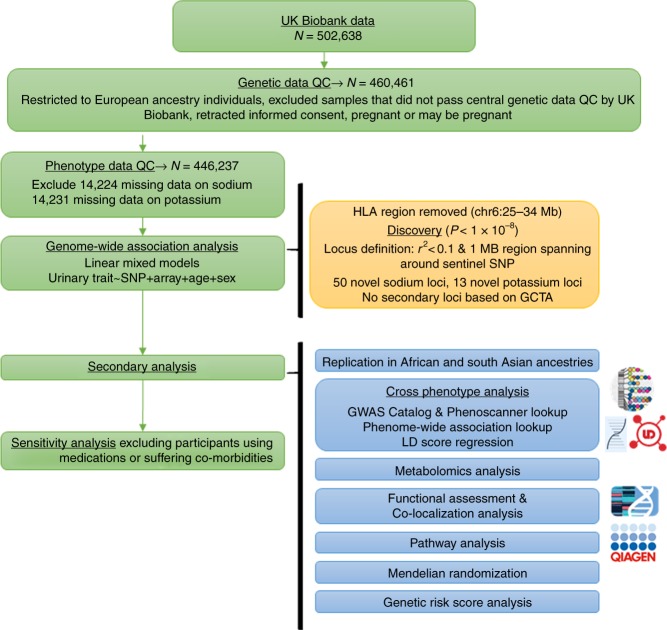
Overview of study design

We performed GWAS of urinary sodium and potassium excretion (from spot urine) using linear mixed model (LMM) association testing implemented in BOLT-LMM (v2.3) software (Fig. 1; Supplementary Fig. 1)^14^. We, included ~8.8 M single-nucleotide polymorphisms (SNPs) imputed to the Haplotype Reference Consortium (HRC) panel at MAF < 0.5% from European ancestry participants in UKB (genotyping and imputation [GRCh37] data release 2017). Characteristics of the population are presented in Supplementary Table 1. Of the 50 novel loci identified for urinary sodium and 13 for urinary potassium excretion, 4 overlapped between sodium and potassium excretion (Supplementary Datas 1–6). Conditional analysis revealed no secondary signal. SNP-based heritability was 6.4% for urinary sodium and 4% for potassium excretion.

The strongest urinary sodium locus was in *MLIP* gene (rs838133) (*P* = 1.9 × 10^−25^) followed by *CYP1A1* (rs2472297) (*P* = 6.7 × 10^−23^) and *FTO* (rs11642015) (*P* = 6.7 × 10^−23^) loci. *MLIP* is a muscular lamin A/C interacting protein with protein binding transcription factor activity. *CYP1A1* encodes a cytochrome P450 superfamily enzyme involved in drug metabolism and lipid synthesis. It is also known for association with habitual coffee intake^15^. *FTO* is associated with body mass index (BMI) and other anthropometric traits^16^. We, additionally, identified SNPs within neuronal sodium channel and potassium channel loci *SCN2A* and *KCD13*, as well as ten microRNA and long intergenic noncoding RNA genes. The strongest urinary potassium signal was at *ADRA2C* (an alpha-2-adrenergic receptor) followed by *CYP1A1* and *AHR* loci; the latter two genes have previously shown association with coffee intake^15^.

Our sensitivity analysis (*n* = 262,531) excluding participants who suffered from renal diseases or participants who used medications which may affect sodium and potassium excretion showed that 17 sodium SNPs annotated to *AHR*, *MLIP*, *CYP1A1*, *ADH1B*, *LINC01114*, *LINC02424*, *RARB*, *LOC105378330*, *DCDC1*, *PKHD1*, *NOVA1*-AS1, *MLXIPL*, *FTO*, *MIR642A*, *GCKR*, *HTR4*, and *SCN2A* and 3 potassium SNPs annotated to *ADRA2C*, *SLC4A7*, *CYP1A1* remained genome-wide significant despite the large reduction in sample size (Supplementary Datas 7 and 8). Our LMM-based results showed that 33 urinary sodium lead SNPs and 8 urinary potassium SNPs remained strongly (*P* < 1 × 10^−5^) associated with urinary traits, the slightly larger *P* value is likely due to the decrease in the sample size as the effect estimates are quite small. Effect estimates of the lead SNPs were correlated (*r* = 0.98) before and after exclusion for urinary sodium. The correlation was 0.97 for urinary potassium SNPs. Genome-wide correlation of effect estimates before and after exclusion was 0.79 for urinary sodium and potassium SNPs.

### Cross phenotype analysis

**Fig. 2:**
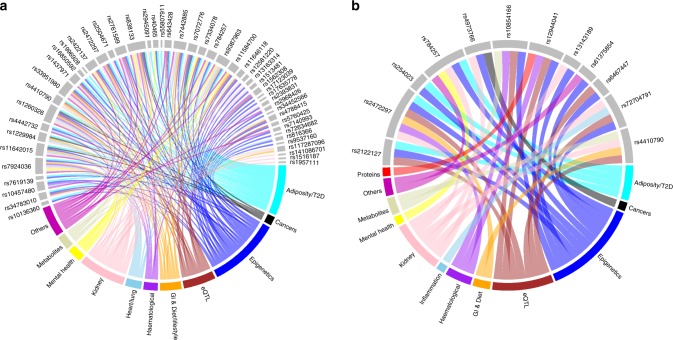
Association of urinary trait loci with other traits. Plots illustrate GWAS Catalog and Phenoscanner associations of **a** urinary sodium excretion genome-wide significant loci with anthropometric traits, lipid, cancers, alcohol, autoimmune diseases, diet, hematological, and neurological diseases. **b** Urinary potassium excretion loci with mainly anthropometric traits, autoimmune diseases, heart and lung diseases, cancers, diet, hematological, and neurological diseases

We examined the association of identified loci with other traits using previously published data included in the GWAS Catalog^17^ and found associations with anthropometric traits as well as inflammation, cancers, diet, lifestyle factors, and hematological traits at *P* < 5 × 10^−8^ (Supplementary Datas 9 and 10; Fig. 2). Look up in GeneATLAS^18^ showed that 45 (90%) urinary sodium loci and 10 (77%) urinary potassium loci were associated with UKB anthropometric traits and/or dietary habits including salt added to food, coffee, fruit, and water intake at *P* < 1 × 10^−6^ (Methods; Supplementary Datas 11 and 12). These findings imply tight co-variation between sodium, dietary choices and BP.

Using LD score regression^19^, we found that urinary sodium and potassium had shared heritable contribution with anthropometric traits, lipoproteins and triglyceride, diabetes, smoking, education and neuroticism (Supplementary Datas 13 and 14). Specifically, we observed a positive shared heritable contribution for urinary sodium excretion with whole body water mass and alcohol consumption frequency (Supplementary Datas 13 and 14). Shared heritable contribution of urinary sodium excretion was negative with red and white wine intake and positive with beer intake (Supplementary Data 13) whereas shared heritable contribution of urinary potassium excretion with wine intake was positive while it was negative with beer intake (Supplementary Data 14). This observation shows importance of loci involved in intake of alcohol subtypes in connection with urinary sodium and potassium excretion.

With regard to pleiotropy with BP, we observed that eight sodium loci were associated with systolic BP (SBP) and ten with diastolic BP (DBP) (Supplementary Datas 15 and 16). The strongest BP SNPs were at *CYP1A1* and *ADH1B* loci (the latter is known for association with alcohol consumption)^20^. SNPs in loci annotated to *MIR588*, *CYP1A1*, *DCDC1*, *DCTPP1*, *and MLIP* were associated both with SBP and DBP. Of the potassium loci, only *SLC4A7* and *CYP1A1* loci were associated with SBP and DBP. *SLC4A7* gene is a co-transporter of sodium/bicarbonate and is known to cause hypertension in model organisms^21^. We, additionally, observed that sodium excretion genetic risk score (GRS) comprising the collective effect of our lead urinary sodium SNPs among the European ancestry subset of the UKB was associated with average annual increase in SBP (beta = 0.06; 95% CI: 0.00–0.11; *P* = 0.02) and DBP (beta = 0.03; 95% CI:0.00–0.06; *P* = 0.03).

### Metabolomics

Metabolomics analysis using plasma samples from Airwave study^22^ (Methods) showed that 12 sodium and potassium loci (Supplementary Fig. 2) were associated with various metabolites including vitamin A, amino acids, carbohydrates, xenobiotics, and major components of lipid and lipoprotein fractions. Two loci at *CYP2A6* (known for its effect on smoking-related behavior^23,24^) and *GCKR* showed association with numerous metabolites. *GCKR* is known to be involved in diabetes risk and metabolism of glucose and lipids^25–28^. It is well established that insulin decreases excretion of sodium in the kidneys^29^. Insulin-stimulated sodium transporters are found all along the nephron, in renal proximal tubule, loop of Helen, distal convoluted tubule, and cortical collecting duct^30^.Our online metabolomics look-up within metabolomics database of Phenoscanner^31^, showed that *GCKR* was associated with mannose (an epimer of glucose), alanine, isoleucine, pyruvate (an alpha-keto acid), and various lipid metabolites. We also observed that *FKBP8* potassium locus was associated with myo-inositol a metabolite of vitamin B complex group (Supplementary Datas 17 and 18, Supplementary Table 2).

### Functional assessment

**Fig. 3:**
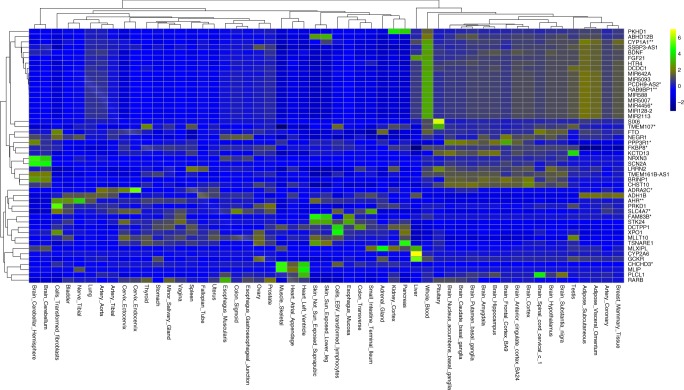
Standardized median gene expressions across 53 tissues for genes mapped to sodium and potassium loci. Genes with no flag are the genes near sodium excretion loci. Genes flagged with one star are genes near potassium excretion lead loci. Genes flagged with two stars are genes near both sodium and potassium excretion lead loci. Blue spectrum color shows low expression, brown spectrum represents moderate expression and green to yellow spectrum show high expression

Using the Genotype-Tissue Expression (GTEx) database^32–34^, we sought to investigate if our lead SNPs are known to have significant expression quantitative trait loci (eQTL) associations. Urinary sodium variants showed eQTL effect on gene expression levels for 20 genes and potassium variants for four genes (Supplementary Table 3, Supplementary Datas 19 and 20). The annotated genes for urinary sodium or potassium loci were mainly expressed in adipose tissue, coronary arteries, and brain (Fig. 3). A few genes showed broad expression profiles across a range of tissues.

**Fig. 4:**
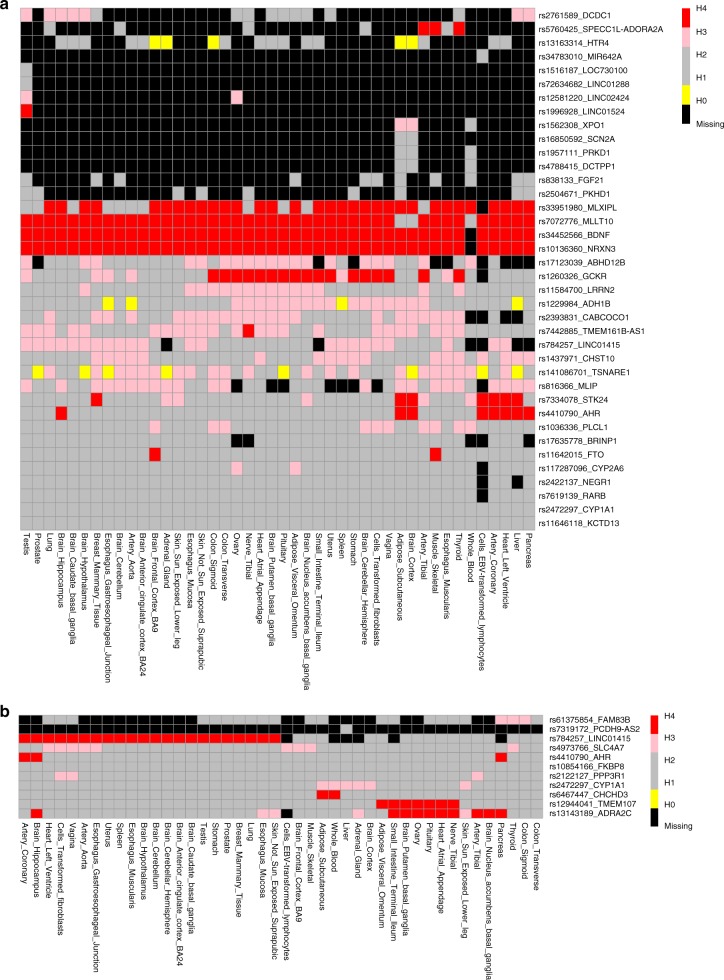
Heat map clustering of co-localization analysis results. GTex-based eQTL results from Ensemble REST API is used between nearest gene to the lead SNPs and each SNP in 200 KB region surrounding lead SNPs. Rows show lead SNP-nearest gene combinations for which eQTL is obtained. Columns show tissues from which eQTL is obtained. If achieved, posterior probabilities >0.75 is colored for H0 (yellow; on association with either trait), H1 (gray; association with urinary trait, not with gene expression), H2 (gray; association with gene expression, not with urinary trait), H3 (pink; association with urinary trait and gene expression, two independent SNPs), and H4 (red; association with urinary trait and gene expression, one shared SNP). Black cells show missing eQTL information. **a** Heat map clustering based on co-localization for urinary sodium loci. **b** Heat map clustering based on co-localization for urinary potassium

Co-localization analysis highlighted seven urinary sodium loci (*MLXIPL*, *MLLT10*, *BDNF*, *NRXN3*, *STK24*, *GCKR*, and *AHR*) and three urinary potassium loci (*LINK01415*, *TMEM107*, and *ADRA2C*) that harbor genetic variants which demonstrate association with both urinary traits and gene expression (Fig. 4).

### Pathway analysis

**Fig. 5:**
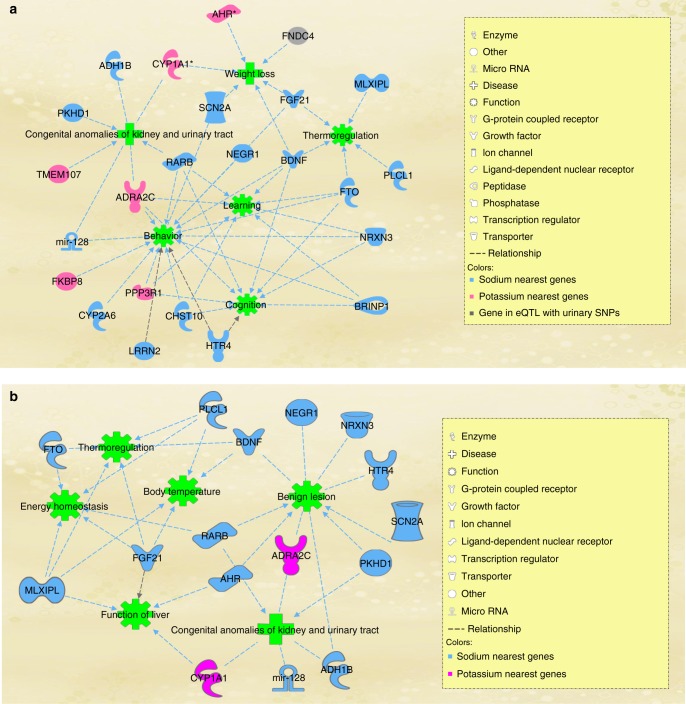
Sodium and potassium loci in behavior, thermoregulation, and weight loss networks. Knowledge-based disease and functional networks connecs urinary trait loci. Analysis and illustration is done using ingenuity pathway analysis software. **a** Analysis using all loci **b** Analysis using loci that remained genome-wide significant after exclusion of participants with known kidney disorders or those using medication

Ingenuity pathway analysis (IPA)^35^ showed that urinary trait loci were enriched in pathways involving behavior (Fig. 5a), followed by congenital anomalies of kidney and urinary tract, weight loss, and thermoregulation (Supplementary Data 21). Behavior was predefined by the IPA as behavioral response to stimuli. It, includes molecular mechanisms and processes that affect, decrease or increase adult behavioral response to stimuli including initiation of behavior or delay in behavior.

We performed a second IPA analysis using loci from our sensitivity analysis excluding individuals under treatment with BP lowering medications, NSAIDS, corticosteroids, and self-reported or hospital records of renal diseases. This exclusive IPA analysis showed that our urinary sodium and potassium loci are involved in thermoregulation, body temperature, energy homeostasis, function of liver, benign lesions, and congenital anomalies of the kidney.

### Mendelian randomization

The list of lead SNPs (*n* = 63) from Supplementary Table 2c and d was used as exposure loci (sodium and potassium excretion SNPs) for MR analyses^36^. The aim was to test effect of sodium and potassium excretion on BP and CVD. We performed MR analysis using effect estimates of sodium and potassium excretion lead SNPs in full UKB sample against effect estimates of these SNPs for BP from the International Consortium for BP (ICBP) results. There is no sample overlap between ICBP and UKB.

In parallel and to test the same hypothesis while ensuring independence of the samples for MR analysis, we divided UKB sample into two independent non-overlapping subsamples, north (*n* = 224,883) and south (*n* = 221,354) (Supplementary Table 4; Supplementary Fig. 3). Combination of UKB north and UKB south subsample is the full UKB sample. We performed additional sensitivity MR analyses where we randomly divided the full UKB sample into two equal sets. Combination of the two random split subsamples is the UKB full sample. For the 63 SNPs from Supplementary Table 2c and d, SNP effect estimates on exposure (urinary traits) in one subsample was obtained and were used against SNP effect estimates on outcome (BP) obtained from the other subsample.

For MR analysis of urinary electrolytes on CHD, we used CARDIOGRAM data from Nikpay et al.^37^ and Nelson et al.^38^. There is no sample overlap between CARDIOGRAM data from Nikpay et al. and UKB.

After removal of outliers using MR-PRESSO (Supplementary Datas 22–25, Supplementary Table 5), inverse variance weighting effect estimates suggested a positive effect of urinary sodium on DBP (Supplementary Data 24; based on UKB random split subsample) and CHD (based on Nelson et al.) as well as an inverse association for urinary potassium on SBP (Supplementary Datas 24–25; Supplementary Table 5; Supplementary Figs. 4–6).

Sensitivity analysis using ICBP data shows that the interpretation of the results remained generally similar compared with UKB split subsamples. The diagnostic plot for the MR effect estimate of urinary potassium on SBP using UKB vs. ICBP outcomes (Supplementary Fig. 6) shows major similarities in distribution of effect estimates. The only difference we observed was a decreased in MR effect estimate of urinary potassium on SBP when we used ICBP results rather than UKB split subsamples (Supplementary Fig. 8). Given that population differences between UKB and BP consortium is much larger than population differences between UKB split subsamples, we believe the decrease in effect estimate for the results of urinary potassium on SBP using UKB vs. ICBP consortium could partially be due to population differences rather than pure effect of urinary electrolytes on BP. Our results also show a caveat for MR that small variation in distribution of effect estimates can have large impact on MR effect estimates as shown in the example above.

We observed large differences between MR-Egger and other MR tests. MR-Egger estimates are less efficient than estimates from other methods and are highly sensitive to violations of the MR assumptions and the validity of the InSIDE assumption in particular (Burgess et al. https://arxiv.org/abs/1606.03729). According to our diagnostic plots, we infer that few outliers in the MR analysis of urinary traits on BP are likely to violate InSIDE assumptions. This leads to a large difference between effect estimate from MR-Egger and effect estimate from other methods. Upon removal of outliers, the remaining instruments are more likely to meet the InSIDE assumption and hence in our analyses, *P* value of MR-Egger increased to a non-significant level in most cases.

We performed additional sensitivity analyses to address the heterogeneity of the association of urinary  sodium loci with BP by performing MR on loci involved in biological pathways based on IPA analysis. MR analysis using loci involved in behavior and thermoregulatory pathways suggested a positive effect for urinary sodium on DBP (behavior pathway loci) and on SBP, and DBP (thermoregulatory pathway loci; Supplementary Data 26).

### Replication in other ancestries

All lead SNPs (i.e., those with lowest association *P* value per locus) identified in discovery stage together with all SNPs in 1 MB region surrounding the lead SNPs if they showed association with urinary traits at *P* < 1 × 10^−8^, were sought for replication among other ancestries in the UKB. SNPs within *DCDC1* locus were replicated (LMM-based *P*_replication_ < 9.8 × 10^−4^) among the UKB African population (*n* = 7612) and SNPs in a region flanked by *MIR-588* and *CENPW* genes replicated in the UKB South Asian population (*n* = 10,095) (Supplementary Data 27). *DCDC1* is in close vicinity (<1 MB) of a potassium channel gene (*KCNA4*) and is not previously known to impact electrolyte levels. We also observed that urinary sodium GRS, which we calculated based on lead SNP effect estimates obtained from the main GWAS analysis, was associated with slight increase in urinary sodium excretion among south Asians (beta = 0.01; *P* = 0.03).

We and others^39,40^ have previously shown that lifestyle factors are risk factors for CHD independent of genetic susceptibility^39,40^. The results here take a step forward to investigate the link between sodium, BP and CHD and highlight that behavioral response to stimuli and thermoregulatory pathway scould potentially be involved. The network illustrated in Fig. 5, involves behavior, learning, cognition, thermoregulation, and weight loss nodes. Next to this information, our phenome-wide association analysis within UKB shows association of sodium SNPs with, e.g., salt added to food and dietary intake of meat, coffee, water, and alcohol. Genetic factors involving behavioral response to stimuli may affect dietary decisions that eventually impact urinary sodium and potassium excretion.

 It is previously observed that a decreased core temperature increases vasoconstriction and consequently increases BP via release of adrenaline^41–46^. Only few epidemiological studies have investigated the effect of thermogenesis on SBP^42,47^. Our study for the first time highlights a potential role for urinary sodium loci involved in thermoregulatory pathways in regulation of BP.

It is possible that some of the urinary sodium and potassium loci could  simultaneously be involved in efficiency of medication and cause changes in urinary electrolyte levels Thus, we performed GWAS sensitivity analysis excluding individuals under treatment with BP lowering medications, NSAIDS, corticosteroids, and self-reported or hospital records of renal diseases. Despite losing half of the sample size, over one-third of sodium loci survived this sensitivity analysis. While the decrease in the number of genome-wide significant loci could be the result of decrease in sample size and losing statistical power, some of the lost sodium loci could in fact follow a more complicated biological mechanism involving use of medications. A dedicated pharmaco-epidemiologic study could provide insight on the interaction between such loci and medication use. Thermoregulatory pathways and pathways involved in congenital anomalies of the kidney remained consistently enriched with urinary sodium and potassium loci in the sensitivity analysis highlighting importance of these biologic function in regulation of urinary sodium and potassium excretion.

Sodium and potassium are vital for cellular function and are typically exchanged between intra- and extra-cellular space using ATP-dependent sodium/potassium pumps, which actively transport sodium in and potassium out of the cell ^48^. The results of our cross-phenotype analyses indicate that urinary trait loci are highly inter-correlated with lipids and anthropometric traits. This is further supported by recent functional evidence that showed direct effect of sodium on lipid accumulation in adipocytes suggesting that pathways involved in regulation of sodium might be directly involved in lipid metabolism^8^.

Evidence from epidemiological studies, animal models and randomized clinical trials have shown a significant, direct association of sodium, and inverse association of potassium intakes with BP^2–4,6,8–10^. Our MR analysis supports this evidence. Our study benefits from a large sample size, access to metabolomics data, MR analysis, complementary application of bioinformatics methods, and extensive exploration of the pleiotropic effects of the identified loci. Limitations of our study include the use of spot urine samples as the only available source to quantify sodium and potassium excretion within UKB, and the reliance on a one stage analysis; to account for this, we used a more stringent empirically calculated significance threshold for genome-wide significance^49^ and tested our results in other ancestries in UKB.

In summary, our study provides 63 novel loci for urinary sodium and potassium excretion with multiple tissues such as brain, adipose tissue and vasculature possibly involved. We showed that genetic underpinning of urinary sodium and potassium excretion is highly pleiotropic reflecting that many pathways might be involved in their regulation. The association of the identified loci with anthropometric measures, lipids, and fluid intake indicates that variation in these urinary traits might involve mechanisms beyond dietary intake of sodium and potassium. The eQTL enrichments in brain and enrichment in functions related to behavioral response to stimuli might imply that the heritable component of urinary sodium and potassium excretion could be driven by behavioral response rather than mechanisms purely involving ion transport in the kidneys, in contrast to what is known about some other urinary measurements such as uric acid^50^.

Our study supports a genetic link between urinary traits, adiposity, BP and CHD. Urinary traits reflect the complex interplay between dietary intake, homeostatic mechanisms that tightly control intra- and extra-cellular concentrations of sodium and potassium excretion via the kidney and other pathways^51^, and potential genetic mechanisms. This complexity complicates the ability to unravel the sequence and direction of the causal pathways involved and deconvolve the inter-dependence between the urinary and other variables (e.g., BMI). In such circumstances, including extensive pleiotropy, current MR methodologies are limited in their ability to provide causal interpretations.

## Methods

### Study design and participants

We used data from the UKB^11–13^ study which includes approximately 500,000 individuals aged 40–69 years from European, African, and Asian ethnicity. North West Multi-Center Research Ethics Committee has approved the UKB study. Approval for this research was obtained from the UK Biobank Research Ethics Committee and Human Tissue Authority, and the participants gave informed consent. We removed from our analysis all participants from the UKB study who withdrew consent.

Study participants were ascertained through United Kingdom National Health Service registers across 22 centers in Great Britain between 2006 and 2010^13^. We included individuals of European ancestry following quality measures and exclusions (sex discordance, high missingness/heterozygosity). Individuals with non-European ancestry were excluded from the main analysis. Allocating individuals to ethnicity groups was based on self-reported ethnicity matched with PCA ancestry clustering using the *kmeans* clustering method. We excluded participants who had withdrawn consent (*n* = 19), as well as those who were pregnant or unsure of their pregnancy status at baseline (*n* = 372). After removing participants of non-European ancestry (*n* = 41,786) and individuals with missing values on urinary sodium (*n* = 14,224) and urinary potassium (*n* = 14,231), 446,237 individuals were left for GWAS of urinary sodium and 446,230 individuals for GWAS of urinary potassium. As part of the study, we performed MR analysis (see below) to investigate potentially causal effects of urinary traits on BP. According to MR methodology, variance of an instrumental variable estimate will be smaller if it is estimated from the same study^52^. When distinct samples are used, bias due to weak instrumental variable shifts results toward the null. In contrast, in a situation where the two samples perfectly overlap, the weak instrument bias shifts the results toward the observational association between the risk factor and outcome^53^. Since our study avoided sample overlap, potential weak-instrument bias is expected to be toward the null. To avoid sample overlap in our MR analysis, we divided the UKB sample into two subsamples of north (*n* = 224,883) vs. south (*n* = 221,354). This was according to the geographic locations where data were initially obtained. This division of samples was designed to ensure geographical independence of the two samples and to minimize sharing of similar environments, which may have not been achieved by random splitting of the sample. To ensure the results were not biased by geographical differences in the UKB sample, we compared our results with an additional MR analysis where we randomly split the full UKB sample into two equal subsamples.

We performed a sensitivity analysis excluding participants who used antihypertensive medication, corticosteroids or NSAIDs as well as those suffering from self-reported kidney disease, or hospital records for diagnosis of renal disease. Diagnoses codes were based on International classification of diseases (ICD 10) codes N00.0–N39.9. The final sample for sensitivity analysis included 262,531 participants of European ancestry.

### Genotyping and imputation

Genotyping and imputation in the UKB have been described in detail elsewhere^54,55^. Briefly, a custom Affymetrix UKB Axiom array (designed to optimize imputation performance) was used for genotyping of DNA samples obtained from the UKB study participants. Imputations in UKB were performed centrally using an algorithm implemented in the IMPUTE2 program. Genetic principal components to account for population stratification were computed centrally by UKB.

### Urinary measurements and BP

Details of quality control and sample preparation for urinary measurements have been published previously by UKB^56^. Sodium and potassium concentrations in stored urine samples were measured by the ion selective electrode method (potentiometric method) using Beckman Coulter AU5400, UK Ltd. Analytic range for sodium and potassium was 2–200 mmol/L, and 10–400 mmol/L, respectively.

Details of calculation of BP values used for this analysis are described in detail elsewhere^39,57^. BP was measured at baseline, after a two-min rest using an appropriate cuff and an Omron HEM-7015IT digital BP monitor. We calculated mean SBP from all available automated or manual reading BP measurements. For individuals who reported taking BP-lowering medication (*n* = 91,648 individuals), we added 15 and 10 mm Hg to SBP and DBP, respectively as has been done previously^58^.

### Genome-wide association analysis

For GWAS of urinary sodium and potassium, we performed LMM association testing implemented in BOLT-LMM (v2.3) software^14^ that corrects for confounding from population structure and cryptic relatedness. We assumed additive genetic model on continuous log transformed spot urinary sodium and potassium excretion adjusted for age and sex. Additional to the QC that was done centrally by the UKB, we applied some filters including MAF > 5%; HWE *P* > 1 × 10^−6^; missingness < 0.015 for the initial modeling step to estimate parameters of the LMM. We restricted the main association analysis to SNPs imputed from the HRC panel (*n* = 39,235,157), of which ~8.8 million SNPs passed MAF ≥ 0.5% and INFO > 0.1 thresholds.

We empirically assessed the correlation between nearby test statistics in order to calculate the number of independent statistical tests^49^ to be used as our pre-set significance threshold (1 × 10^−8^). We removed all SNPs in HLA region (chr6:25–34 MB). In order to shortlist the lead SNPs, association tests passing this pre-set permutation-driven threshold were ranked in order of significance with stronger associations locating at the top of the list. We then removed all SNPs in the region of ±500 kb surrounding the best ranking SNPs. We continued this approach until there were no SNPs that overlapped within ±500 kb region. We consequently LD pruned the final list of SNPs using UKB individual level data with LD threshold of *r*^2^ ≥ 0.1 using PLINK2 software^59,60^. To detect secondary signals, we used the urinary sodium and potassium GWAS summary-level data and performed conditional analysis using the Genome-wide Complex Traits Analysis (GCTA) software^61^. Our criteria for selection of secondary signals included MAF ≥ 0.5% and GCTA based *P* < 1 × 10^−8^. We used genome-wide conditional analysis with stepwise model selection as well as locus specific conditional analysis for urinary traits conditioned on the lead SNPs within each locus.

To investigate the effect of lead SNPs on urinary traits among other ancestries, we performed association analysis on all the SNPs in 1 MB region surrounding the lead SNPs if they showed association with urinary traits at *P* < 1 × 10^−8^. We used BOLT-LMM regression among 10,095 individuals of south Asian and 7612 individuals of African ancestry within UKB. We considered statistical significance if the SNPs passed *P* value threshold of 9.8 × 10^−4^.

### GRS analysis

We calculated weighted sodium and potassium excretion GRS based on the effect sizes of the final sentinel variants identified in the main UKB GWAS analysis. We then investigated association of this GRS with urinary sodium and potassium excretion among African and South Asian populations in UKB. We also investigated the association of GRS with average annual change in BP at first repeat assessment visit (2012–2013) in the European subset of the UKB study compared with baseline assessment. To obtain change in BP values, we subtracted values obtained for BP at baseline from values obtained at first repeat assessment divided by the time difference in years between baseline and first repeat assessments. For this analysis individuals with missing values for repeat BP measurement and individuals with first and second degree relationships were excluded. The final analysis on 19,686 individuals was adjusted for age, age^2^ and sex.

### Phenome-wide association analysis

We looked-up phenome-wide association analysis (Phewas) results for urinary trait lead SNPs within GeneATLAS^18^ (2018 version) performed on the 452,264 individuals and 778 phenotypes from the UKB. To claim significance, we used a Bonferroni corrected significance threshold of *P* < 1 × 10^−6^, Bonferroni corrected for the number of lead SNPs multiplied by 778 phenotypes.

### Pleiotropy investigation

To investigate shared heritable contribution between urinary traits with other phenotypes, we used the Broad institute LD hub^62^ tool to perform LD score regression analysis using GWAS summary statistics data as well as calculation of SNP-based heritability. To investigate trait pleiotropy and evidence of association of urinary trait loci with other traits, we extracted from the GWAS Catalog^17^ all urinary sodium and potassium associated SNPs at *P* < 1 × 10^−8^ in 1 MB region surrounding the lead SNPs.

### Metabolomics

To study the metabolomics signature of our lead SNPs, we used data from the Airwave Health Monitoring Study (Airwave)^22^, a cohort of UK police forces. The Airwave study includes metabolomic data generated by Metabolon platform on 1967 plasma samples, including 1048 mostly identified metabolites and some unidentified species^63^. In addition, we analyzed serum metabolomics data from 2022 serum samples in which 105 lipoprotein subclasses were quantified by the Bruker IVDr LIpoprotein Subclass Analysis (B.I.-LISA; Bruker Biospin, Rheinstetten) (https://www.bruker.com). We computed significance thresholds using an estimated 5% family-wise error rate using a permutation approach (10.1021/pr1003449 and 10.1021/acs.jproteome.7b00344). We performed association tests with metabolomics data using Spearman partial correlation analysis adjusted for age and sex and genetic principal components.

We additionally used PhenoScanner^31^, a publicly available data set of SNP-metabolites associations to obtain more information regarding metabolomics signatures of urinary traits loci.

### Functional assessment

We used multiple genomic tools to investigate functional impact of the lead loci. We annotated SNPs to the nearest gene within the distance of ±500 kB using University of California Santa Cruz (UCSC) genome-browser. We checked functional annotation of the lead SNPs using Variant Effect Predictor (VEP) tool^64^. VEP provides a report of SNP characteristics (e.g., intronic and noncoding transcript exon), and SIFT/PolyPhen based functional impact of amino acid substitution. We evaluated all genome-wide significant SNPs for evidence of eQTL using the GTEx database^32–34^(www.gtexportal.org). We additionally searched median gene expression levels in 53 tissues from the GTEX database and standardized gene expression values to map tissue-specific expression of genes near and/or in eQTL (eGenes) with urinary trait lead loci.

### Co-localization analysis

To check if the loci we identified associated with urinary sodium and potassium are the same loci affecting gene expression, we performed a co-localization analysis using *coloc*, a package running under R. To obtain eQTL data for each SNP and gene combination per tissue, we used ensemble REST API^65^. Co-localization analysis was done for each tissue and gene region combination separately. The analysis provides posterior probabilities for H0 (no association with either trait), H1 (association with urinary trait, not with gene expression), H2 (association with gene expression, not with urinary trait), H3 (association with urinary trait and gene expression, two independent SNPs), and H4 (association with urinary trait and gene expression, one shared SNP). We drew clustered heatmaps on the results of co-localization analysis to illustrate SNPs and genes that are similar with regard to tissue-based co-localization.

### Pathway analysis

We performed gene-based variant effect analysis considering direct and indirect relationships within IPA^35^ software (IPA^®^, QIAGEN Redwood City) to evaluate prior knowledge on sodium and potassium loci and to identify over-representation of sodium and potassium loci in various disease and functional pathways. We used the list of nearest genes and genes in eQTL with sodium and potassium loci within IPA and performed variant effects analysis. Out of the most significantly enriched diseases and functions in IPA’s knowledge base, we selected the most statistically significant functions that included at least three genes and collectively covered all urinary trait loci. We then used “displayed as network” function within IPA to illustrate networks connecting our urinary trait loci, using IPA’s organic design.

In IPA, the *P* value of overlap states the statistical significance of the enrichment of a biological attribute (e.g., Canonical Pathway, Upstream Analysis, etc.) in user's dataset.  It compares the proportion of input molecules (e.g., genes) that are associated with a particular biological attribute to the proportion of molecules that we expect to see if the dataset was made up of randomly selected molecules. It is calculated using the right-tailed Fisher's exact test. The *P* value less than 0.05 or (−log *P* value = 1.3) is considered significant by IPA. The smaller the *P* value, the less likely that the association is random and the more significant the association^66^.

### MR analysis

We tested the effect of sodium and potassium on BP and CHD using MR analysis implemented in R package^67^. To avoid sample overlap for exposure and outcome, we divided the UKB sample into two samples according to geographic locations where data were initially obtained (north vs. south). We then performed GWAS investigations in each sample separately. We looked-up the lead SNPs for association with exposure (urinary traits) in northern UKB sample (*n* = 224,883). We subsequently looked-up the lead SNPs for association with outcome (SBP and DBP) within the southern UKB sample (*n* = 221,354). SNP-exposure effects were used as instruments against SNP-outcome effects to assess potential causal effect of urinary traits on BP. In separate MR analysis, we additionally used SNP-BP effect esimates from ICBP. To assess potential causal effect of urinary traits on CHD, we obtained SNP-exposure effects from the full UKB sample and SNP-outcome effects from CARDIOGRAM 1000G CHD GWAS results (CARDIOGRAMplusC4D) from Nikpay et al.^37^ as well as meta-analysis of UK Biobank SOFT CAD GWAS (interim release) with CARDIoGRAMplusC4D 1000 Genomes-based GWAS from Nelson et al.^38^.

For statistical analysis of MR, in the presence of heterogeneity, we used the inverse variant weighting (IVW) method assuming random effect. As sensitivity analysis, we used estimator from the weighted median method^68^ that provides a robust estimate even when up to 50% of the SNPs are not valid for MR. An important assumption of MR is absence of horizontal pleiotropy that occurs when the effect of SNP on outcome is independent of exposure which can be tested using the MR-Egger method^69^. If MR-Egger indicated presence of pleiotropy we repeated the analysis after removing outliers. To detect outliers and correct for widespread horizontal pleiotropy, we used MR Pleiotropy Residual Sum and Outlier (MR-PRESSO) test^70^. In MR analysis using IVW test, we used *P* < 0.008 as our statistical significance threshold taking into account multiple testing for two MR exposures and three different outcomes analyzed.

Cherry picking has been considered as a potential limitation of heterogeneity-based outlier removal methods. It is also noted that outlier removal improves statistical power by reducing noise in estimation whilst a drawback is that the method could increase type 1 error rate resulting in detecting causal effects that are not true. It is necessary to emphasize that any causal conclusion based on our MR results should be inferred with caution, though our MR analysis is supported by previous RCTs that have shown that short-term reduction in sodium intake decreases BP.

### URLs

For GTEx, see www.gtexportal.org. For IPA, see www.qiagen.com/ingenuity. For PhenoScanner, see http://www.phenoscanner.medschl.cam.ac.uk (Phenoscanner integrates results from the GWAS catalog: https://www.ebi.ac.uk/gwas/ and GRASP: https://grasp.nhlbi.nih.gov/). For GeneATLAS, see http://geneatlas.roslin.ed.ac.uk.

### Reporting summary

Further information on research design is available in the Nature Research Reporting Summary linked to this article.

## Supplementary information

Supplementary Dataset 1

Supplementary Dataset 2

Supplementary Dataset 3

Supplementary Dataset 4

Supplementary Dataset 5

Supplementary Dataset 6

Supplementary Dataset 7

Supplementary Dataset 8

Supplementary Dataset 9

Supplementary Dataset 10

Supplementary Dataset 11

Supplementary Dataset 12

Supplementary Dataset 13

Supplementary Dataset 14

Supplementary Dataset 15

Supplementary Dataset 16

Supplementary Dataset 17

Supplementary Dataset 18

Supplementary Dataset 19

Supplementary Dataset 20

Supplementary Dataset 21

Supplementary Dataset 22

Supplementary Dataset 23

Supplementary Dataset 24

Supplementary Dataset 25

Supplementary Dataset 26

Supplementary Dataset 27

Supplementary Information

Reporting Summary

Description of Additional Supplementary Files

## Data Availability

Summary statistics will be made available through the NHGRI-EBI GWAS Catalog https://www.ebi.ac.uk/gwas/downloads/summary-statistics.
