# Supplementary material for: GWAS for urinary sodium and potassium excretion highlights pathways shared with cardiovascular traits
Source: Nat Commun. 2019 Aug 13;10:3653. doi: 10.1038/s41467-019-11451-y (PMC6692500; doi:10.1038/s41467-019-11451-y)
Supplement: Supplementary file 28 — Supplementary Information [file 41467_2019_11451_MOESM28_ESM.pdf]

GWAS for urinary sodium and potassium excretion highlights pathways shared with cardiovascular traits

Pazoki et al.

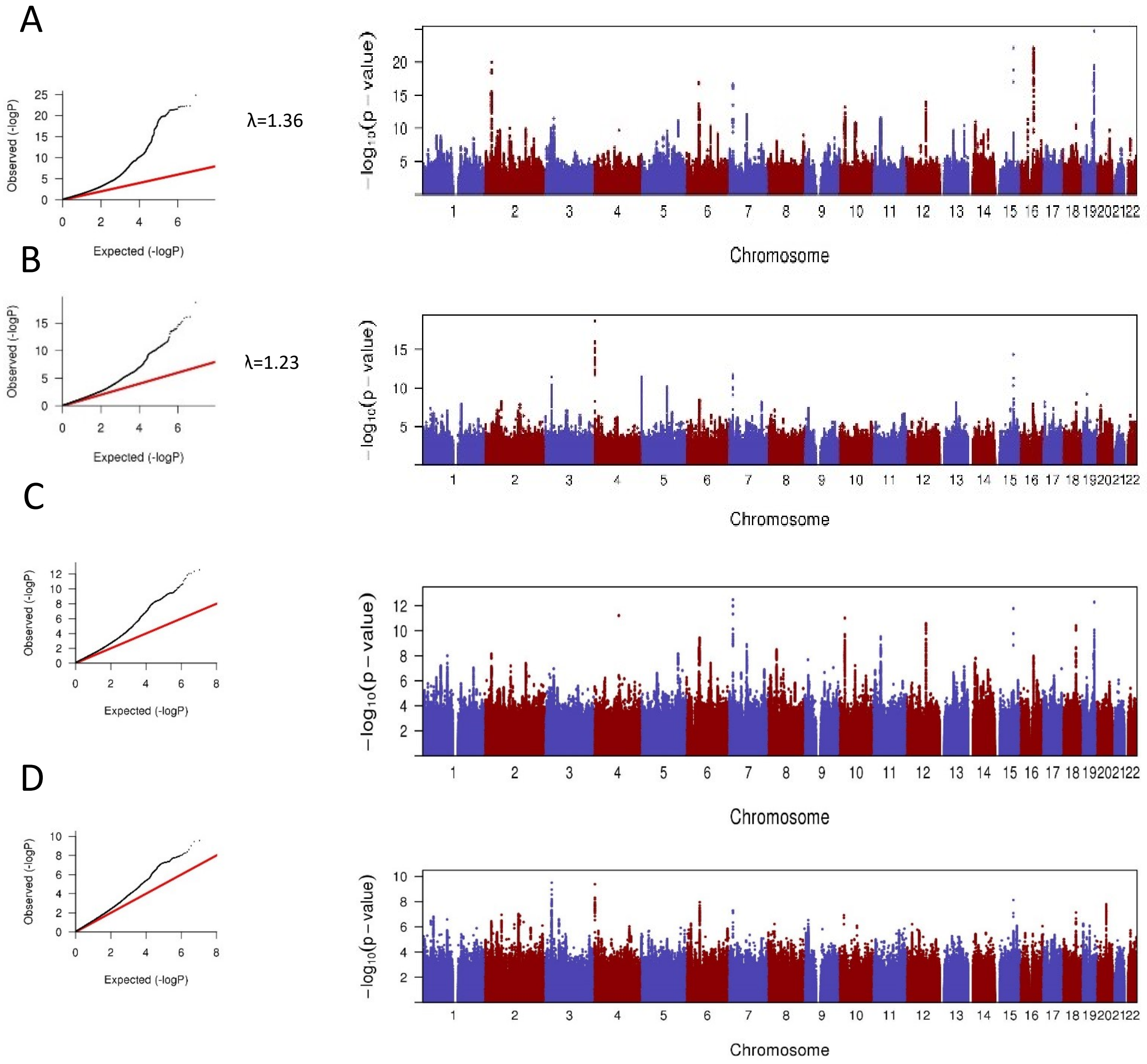

Supplementary Figure 1- Manhattan and Q-Q plots for GWAs of urinary traits. A) urinary sodium GWAS; B) urinary potassium GWAS; C) Urinary Sodium GWAS after medication and renal disease exclusions; D) Urinary potassium GWAS after medication and renal disease exclusions.

Supplementary Figure 2- Association of Urinary trait loci with targeted metabolites in Airwave sample.

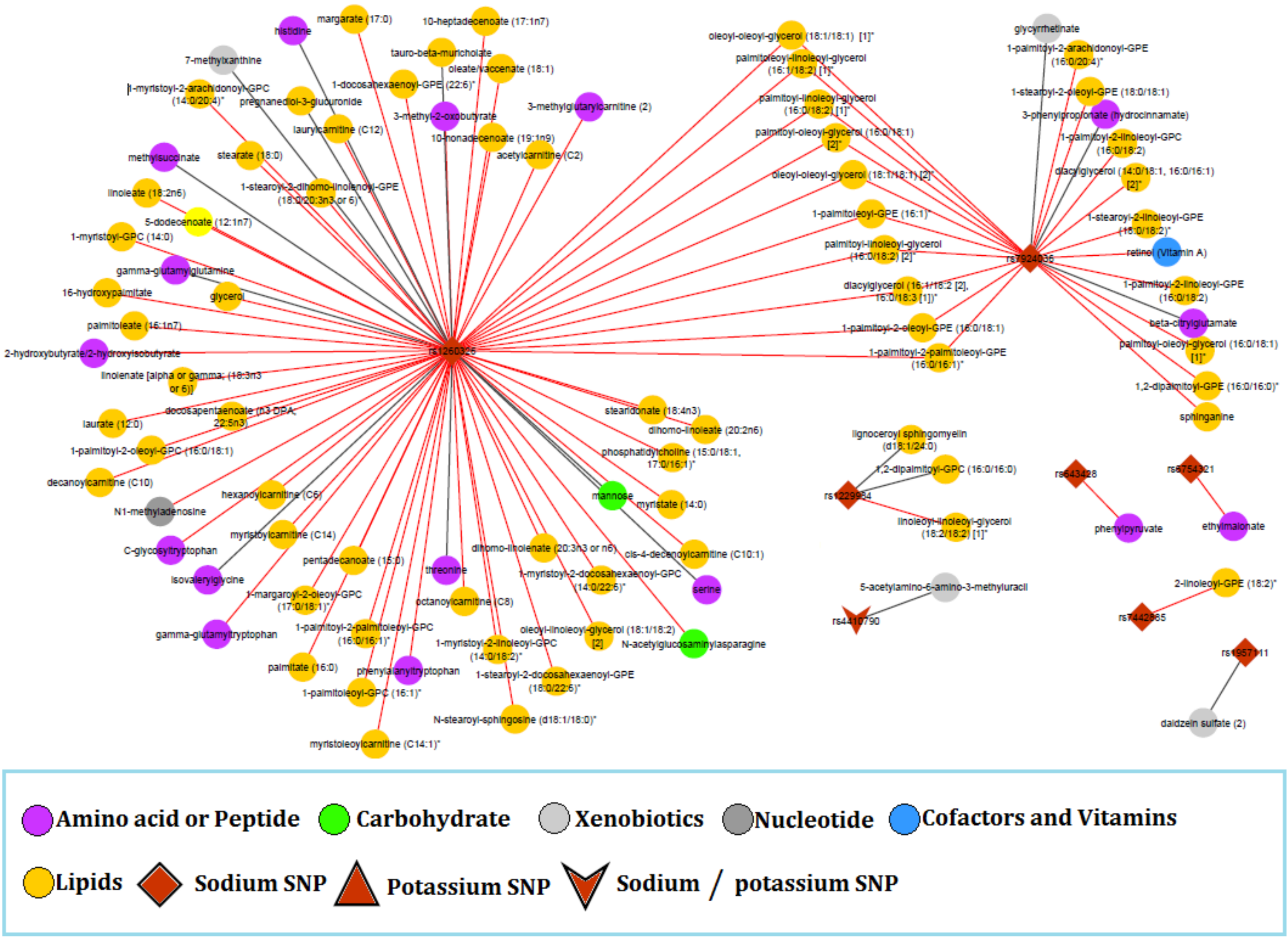

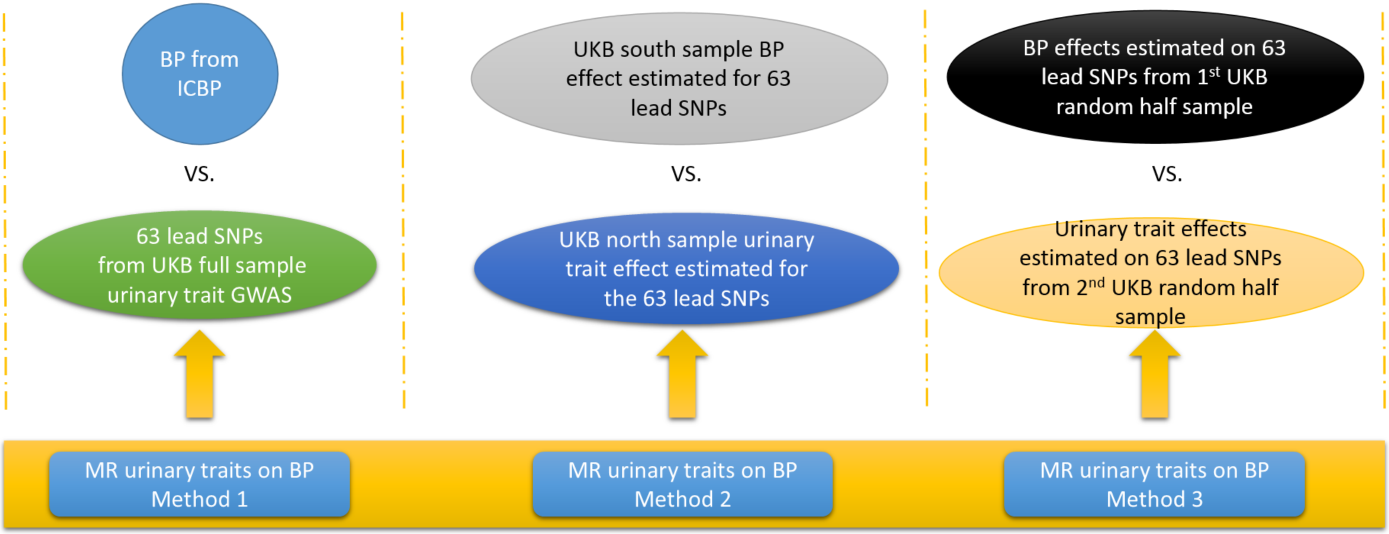

Supplementary Figure 3- Overview of parallel MR approaches for the link between urinary traits and BP. Method 1 represents MR analysis when 63 lead SNPs with effect estimates for urinary traits from the full UK biobank(UKB) sample were used against effect estimates of BP from International Consortium for Blood Pressure (ICBP). ICBP and UKB have no sample overlap. Method 2 represents MR analysis when the 63 lead SNPs with effect estimates for urinary traits from UKB north sample (n=224,883) were used against effect estimates for BP from UKB south sample (n=221,354). These two samples had no sample overlap. Method 3 represents MR analysis when the 63 lead SNPs with effect estimates for urinary traits from 1st UKB random split half sample were used against effect estimates for BP from the second UKB random split half sample. These two samples had no sample overlap.

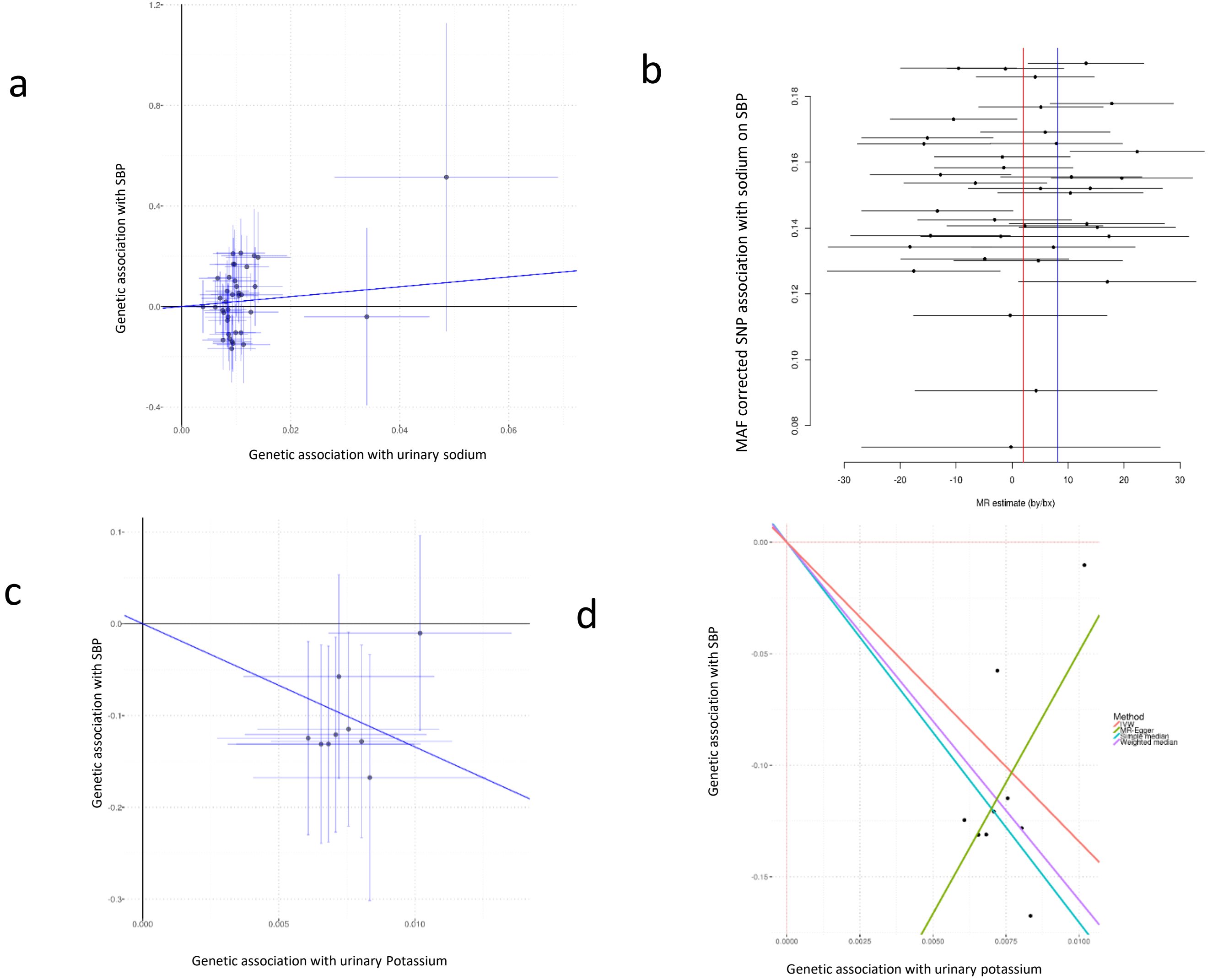

Supplementary Figure 4- Mendelian randomization analysis assessing effect of urinary electrolytes on SBP. a) plot shows the effect size of urinary sodium variants (x axis) against effect size of the same variants for SBP (y axis). b) Funnel plot compares effect estimate from IVW test (blue line) against effect estimate from MR-Egger test (Red line). c) plot shows the effect size of urinary potassium variants (x axis) against effect size of the same variants for SBP (y axis). d) MR plot compares effect estimate from IVW test (red line), simple median (blue line), and weighted median (purple line) against effect estimate from MR-Egger test (green line). The results are based on exposure and outcome effect estimates from UK Biobank north vs. south sample (see methods). SBP, systolic blood pressure; MAF, minor allele frequency; SNP, single nucleotide polymorphism. Error bars refer to 95% confidence interval for the effect estimate.

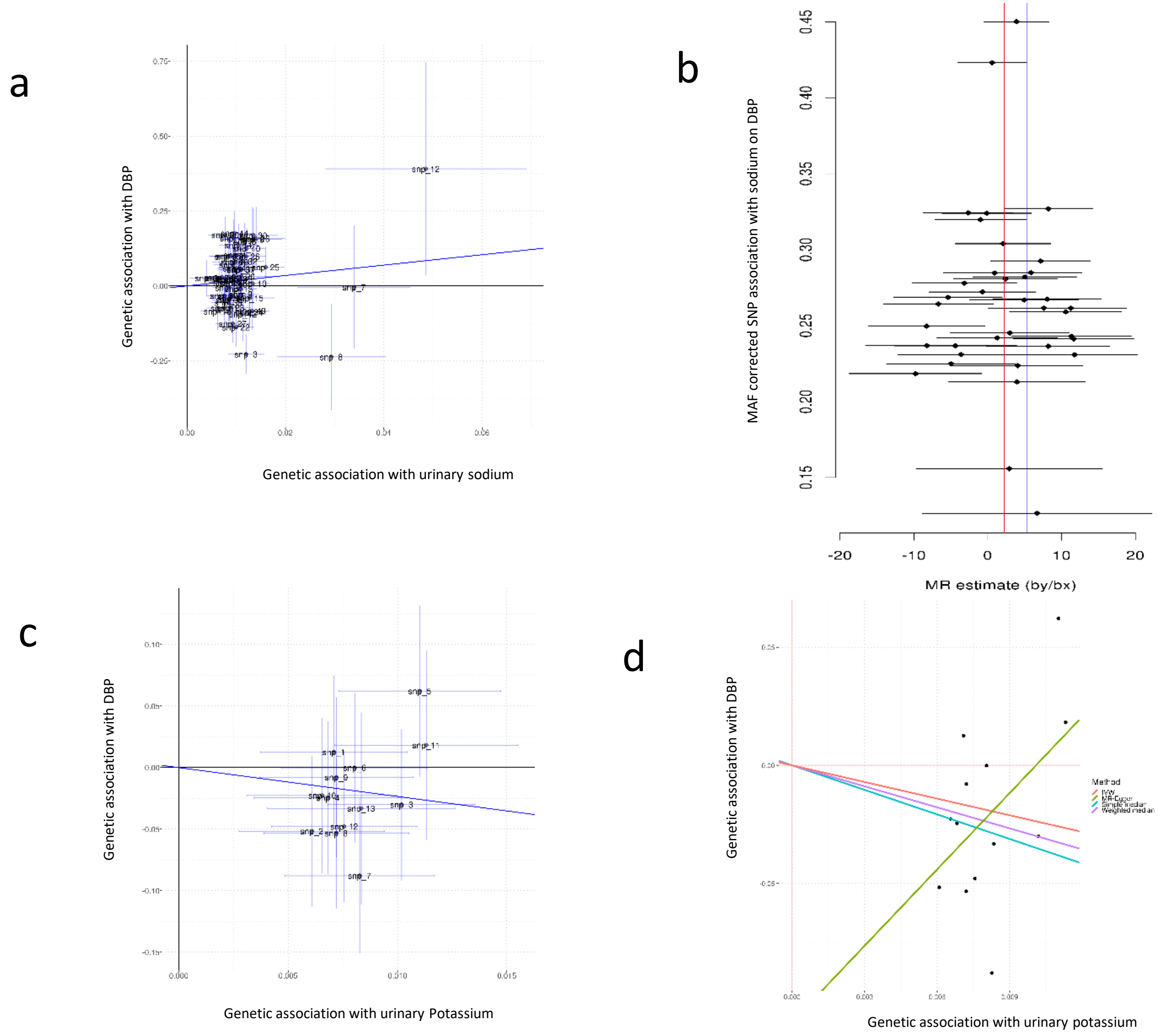

Supplementary Figure 5- Mendelian randomization analysis assessing effect of urinary electrolytes on DBP. a) plot shows the effect size of urinary sodium variants (x axis) against effect size of the same variants for DBP (y axis). b) Funnel plot compares effect estimate from IVW test (blue line) against effect estimate from MR-Egger test (Red line). c) plot shows the effect size of urinary potassium variants (x axis) against effect size of the same variants for DBP (y axis). d) MR plot compares effect estimate from IVW test (red line), simple median (blue line), and weighted median (purple line) against effect estimate from MR-Egger test (green line). The results are based on exposure and outcome effect estimates from UK Biobank north vs. south sample (see methods). DBP, diastolic blood pressure. MAF, minor allele frequency; SNP, single nucleotide polymorphism. Error bars refer to 95% confidence interval for the effect estimate.

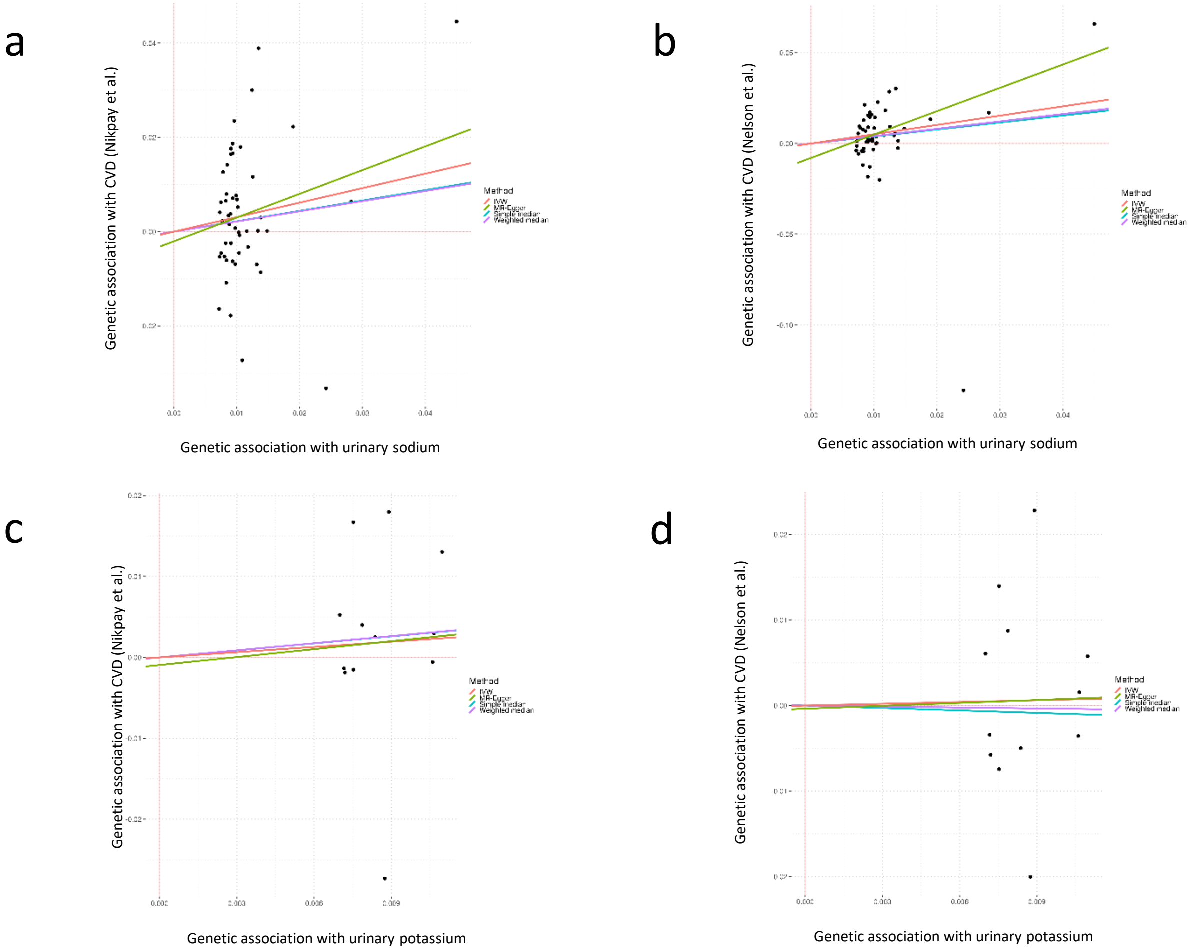

Supplementary Figure 6- Mendelian randomization analysis assessing effect of urinary electrolytes on CVD. a) plot shows the effect size of urinary **sodium** variants (x axis) against effect size of the same variants for CVD from CARDIOGRAM with **no overlap** with UK Biobank data (y axis). b) plot shows the effect size of urinary **sodium** variants (x axis) against effect size of the same variants for CVD from CARDIOGRAM with overlap with UK Biobank data (y axis). c) plot shows the effect size of urinary **potassium** variants (x axis) against effect size of the same variants for CVD from CARDIOGRAM with **no overlap** with UK Biobank data (y axis). d) plot shows the effect size of urinary **potassium** variants (x axis) against effect size of the same variants for CVD from CARDIOGRAM with overlap with UK Biobank data (y axis). The results are based on exposure and outcome effect estimates from UK Biobank vs. CARDIOGRAM (Nikpay et al. or Nelson et al. see methods). CVD, cardiovascular disease.

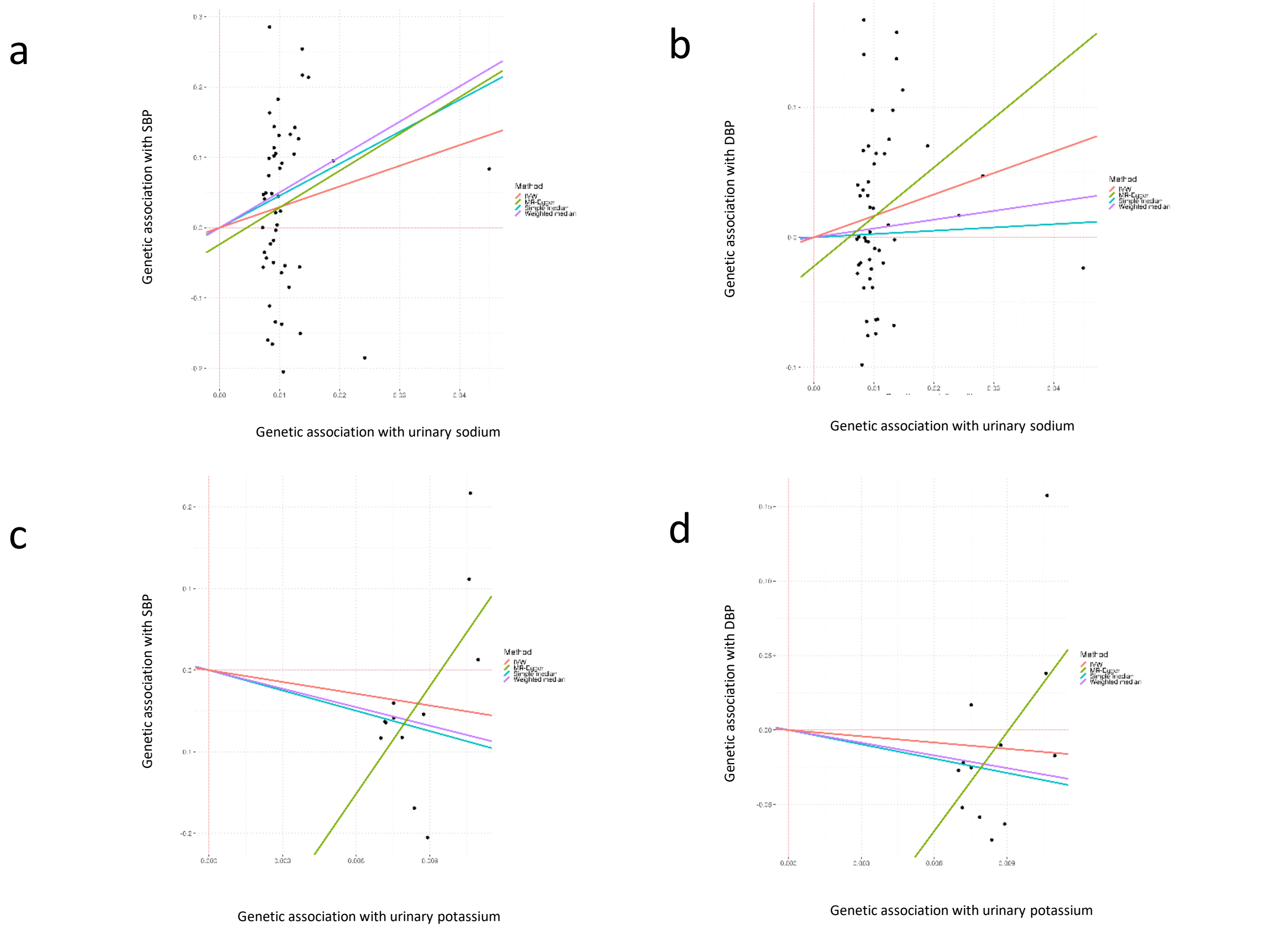

Supplementary Figure 7- Mendelian randomization analysis assessing effect of urinary electrolytes on blood pressure using data from International Consortium for Blood Pressure (ICBP). a) plot shows the effect size of urinary sodium variants (x axis) against effect size of the same variants for SBP (y axis). b plot shows the effect size of urinary sodium variants (x axis) against effect size of the same variants for DBP (y axis). c) plot shows the effect size of urinary potassium variants (x axis) against effect size of the same variants for SBP (y axis). d) plot shows the effect size of urinary potassium variants (x axis) against effect size of the same variants for DBP (y axis). SBP, systolic blood pressure. DBP, diastolic blood pressure.

A) Potassium ~ SBP (UKBN vs UKBS) **B) Potassium ~ SBP (UKB vs. ICBP)**

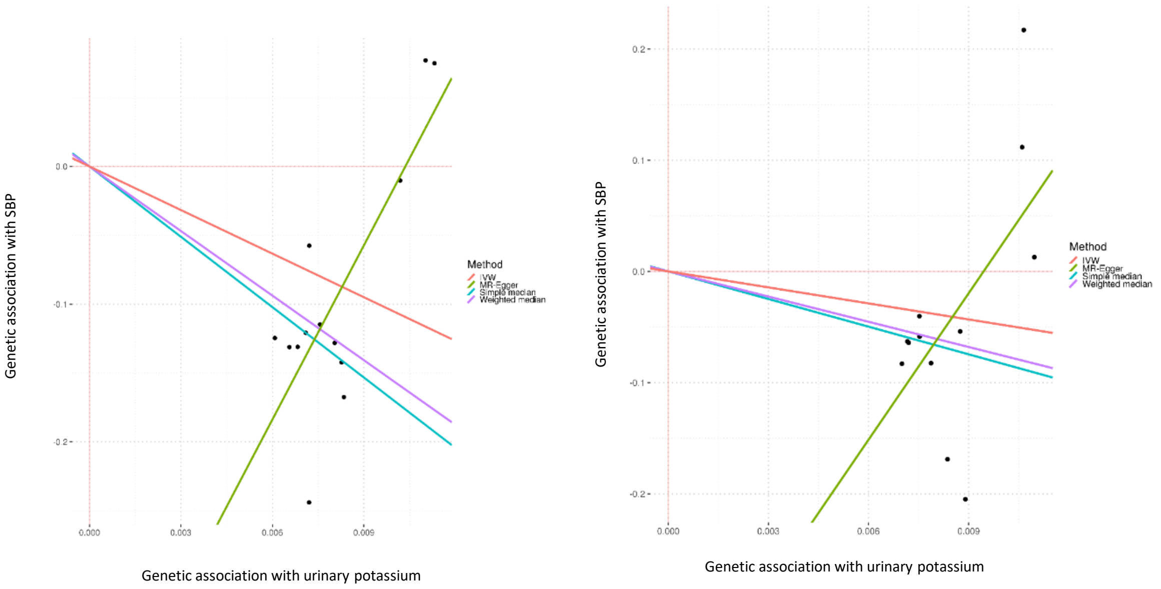

IVW estimate (red line)= -10.56

IVW estimate (red line)= -4.8

Supplementary Figure 8- Comparison of Mendelian randomization analysis assessing effect of urinary potassium excretion pathways on blood pressure using data from UK Biobank (UKB) splits vs. International Consortium for Blood Pressure (ICBP). a) plot shows the effect size of urinary potassium variants (x axis) against effect size of the same variants for SBP (y axis) from UKB north vs UKB South . b) plot shows the effect size of urinary potassium variants (x axis) against effect size of the same variants for SBP (y axis) from UKB vs. ICBP data. SBP, systolic blood pressure.

Supplementary Table 1: Baseline characteristics of the popoulation for analysis.

Characteristics	Urinary sodium sample (n=446,237)	Urinary potassium sample(n=446,230)
Age-yr	56.8(8)	56.8(8)
Male sex -no.(%)	205060(45.95)	204824(45.9)
Hypertension-no./total no(%)	225368(54.23)	225334(54.22)
HTN treatment-no./total no(%)	91648(20.54)	91681(20.55)
Lipid treatment-no./total no(%)	76949(17.24)	76945(17.24)
Diabetes mellitus-no./total no(%)	21290(4.78)	21312(4.79)
Body mass index	27.4(4.8)	27.4(4.8)
sedentary lifestyle	4.5(2.5)	4.5(2.5)
current smoking-no.(%)	46273(10)	46245(10)
past smoking-no.(%)	158310(36)	158286(36)
never smoking-no.(%)	239811(54)	239852(54)
Alcohol intake- mean (SD)- gr/day	17.5(21)	17.5(21)
Systolic blood pressure-mean (SD)- mmHg	141.1(20.7)	141.1(20.7)
Diastolic blood pressure- mean (SD)- mmHg	84.3(11.3)	84.3(11.3)
Incident Coronary Artery Disease- N (%)	25680(5.83)	25700(5.84)
Incident Myocardial Infarction- N (%)	11322(2.56)	11325(2.56)
Incident Stroke-N(%)	5703(1.29)	5718(1.29)

Supplementary Table 2: Association of urinary sodium and potassium loci with metabolites in Airwave using ^{1}H NMR B-LISA (serum samples).

SNP	Value	Chr_position_A2_A1	EA	ID	Component	Analyte	rho_beta	pqval_fdr	Locus
rs1260326	0.001463657	Chr2_27730940_T_C	C	TPTG	Total Plasma	Triglycerides	-0.079945424	0.000323468	GCKR
rs1260326	0.001463657	Chr2_27730940_T_C	C	TPCH	Total Plasma	Cholesterol	-0.089382593	5.77506E-05	GCKR
rs1260326	0.001463657	Chr2_27730940_T_C	C	TPFC	Total Plasma	Free Cholesterol	-0.087978414	7.54572E-05	GCKR
rs1260326	0.001463657	Chr2_27730940_T_C	C	VLTG	VLDL	Triglycerides	-0.079964895	0.000322378	GCKR
rs1260326	0.001463657	Chr2_27730940_T_C	C	VLCH	VLDL	Cholesterol	-0.080560971	0.000290623	GCKR
rs1260326	0.001463657	Chr2_27730940_T_C	C	VLFC	VLDL	Free Cholesterol	-0.088291833	7.11088E-05	GCKR
rs1260326	0.001463657	Chr2_27730940_T_C	C	VLPL	VLDL	Phospholipids	-0.080527949	0.000292303	GCKR
rs1260326	0.001463657	Chr2_27730940_T_C	C	VLAB	VLDL	Apo-B	-0.073962093	0.00088147	GCKR
rs1260326	0.001463657	Chr2_27730940_T_C	C	IDFC	IDL	Free Cholesterol	-0.082577201	0.000203596	GCKR
rs1260326	0.001463657	Chr2_27730940_T_C	C	V2TG	VLDL-2	Triglycerides	-0.072580005	0.001100285	GCKR
rs1260326	0.001463657	Chr2_27730940_T_C	C	V4TG	VLDL-4	Triglycerides	-0.087048688	8.98822E-05	GCKR
rs1260326	0.001463657	Chr2_27730940_T_C	C	V4FC	VLDL-4	Free Cholesterol	-0.080500828	0.000293689	GCKR
rs1260326	0.001463657	Chr2_27730940_T_C	C	V5TG	VLDL-5	Triglycerides	-0.083073708	0.000186286	GCKR
rs1260326	0.001463657	Chr2_27730940_T_C	C	V5FC	VLDL-5	Free Cholesterol	-0.073128398	0.00100806	GCKR
rs1260326	0.001463657	Chr2_27730940_T_C	C	V6PL	VLDL-6	Phospholipids	-0.093822182	2.41667E-05	GCKR
rs1260326	0.001463657	Chr2_27730940_T_C	C	L5CH	LDL-5	Cholesterol	-0.074823144	0.000766312	GCKR
rs1260326	0.001463657	Chr2_27730940_T_C	C	L5FC	LDL-5	Free Cholesterol	-0.079592054	0.00034386	GCKR
rs1260326	0.001463657	Chr2_27730940_T_C	C	L5PL	LDL-5	Phospholipids	-0.074221465	0.000845196	GCKR
rs1260326	0.001463657	Chr2_27730940_T_C	C	L6TG	LDL-6	Triglycerides	-0.09238052	3.22057E-05	GCKR
rs1260326	0.001463657	Chr2_27730940_T_C	C	L6CH	LDL-6	Cholesterol	-0.079596053	0.000343623	GCKR
rs1260326	0.001463657	Chr2_27730940_T_C	C	L6FC	LDL-6	Free Cholesterol	-0.074390307	0.000822332	GCKR
rs1260326	0.001463657	Chr2_27730940_T_C	C	L6PL	LDL-6	Phospholipids	-0.08535846	0.000122999	GCKR
rs1260326	0.001463657	Chr2_27730940_T_C	C	L6AB	LDL-6	Apo-B	-0.082842731	0.000194159	GCKR
rs838133	0.001423107	Chr19_49259529_A_G	G	LDTG	LDL	Triglycerides	-0.080580863	0.000289616	MLIP
rs838133	0.001423107	Chr19_49259529_A_G	G	L5TG	LDL-5	Triglycerides	-0.076708662	0.000561166	MLIP
rs838133	0.001423107	Chr19_49259529_A_G	G	L6TG	LDL-6	Triglycerides	-0.09529881	1.79317E-05	MLIP
rs33951980	0.001355076	Chr7_73029437_C_T	T	V3TG	VLDL-3	Triglycerides	-0.072598041	0.001097131	MLXIPL
rs33951980	0.001355076	Chr7_73029437_C_T	T	V3CH	VLDL-3	Cholesterol	-0.071473148	0.001310617	MLXIPL
rs33951980	0.001355076	Chr7_73029437_C_T	T	V3FC	VLDL-3	Free Cholesterol	-0.071959131	0.001214075	MLXIPL
rs33951980	0.001355076	Chr7_73029437_C_T	T	V3PL	VLDL-3	Phospholipids	-0.07254954	0.00110563	MLXIPL
rs33951980	0.001355076	Chr7_73029437_C_T	T	H4TG	HDL-4	Triglycerides	-0.079873604	0.000327519	MLXIPL

EA: Effect allele; rho_beta: effect estimate

Supplementary Table 3- Number of eGenes per tissue for urinary trait loci from eQTL look up using GTeX.

Urinary sodium loci

Tissue	Gene
Thyroid	8
Esophagus_Mucosa	6
Whole_Blood	5
Lung	4
Adipose_Subcutaneous	3
Muscle_Skeletal	3
Artery_Tibial	2
Heart_Left_Ventricle	1
Nerve_Tibial	1
Skin_Sun_Exposed_Lower_leg	1
Stomach	1

Urinary potassium loci

Tissue	Gene
Artery_Tibial	2
Esophagus_Muscularis	1
Whole_Blood	1

Supplementary Table 4: Regions of the UK Biobank study.

Region	Category	Count
UK Biobank North	Bury	28335
	Cheadle (imaging)	19843
	Cheadle (revisit)	20346
	Edinburgh	17201
	Glasgow	18651
	Leeds	44209
	Liverpool	32818
	Manchester	13940
	Middlesborough	21289
	Newcastle	37008
	Newcastle (imaging)	2996
	Sheffield	30397
	Stockport (pilot)	3798
UK Biobank Middland	Birmingham	25503
	Cardiff	17882
	Nottingham	33877
	Stoke	19440
	Swansea	2281
	Wrexham	649
UK Biobank South	Barts	12583
	Bristol	43014
	Croydon	27385
	Hounslow	28879
	Oxford	14062
	Reading	29416

Supplementary Table 5: Comparison of Mendelian Randomization analysis for the potential causal effect of urinary electrolytes on BP and CVD using International Consortium for Blood Pressure (ICBP) SNP-BP association results (at P<0.008).

MR analysis using UKB North vs. UKB South (Original analysis) including all SNPs					
Method	Estimate	Standard Error	95% CI		P-value
IVW estimate for sodium~ SBP UKBN vs. UKBS	1.84	2.09	-2.24	5.93	0.38
IVW estimate forsodium~ DBP UKBN vs. UKBS	1.73	1.27	-0.75	4.21	0.17
IVW estimate for potassium~ SBP UKBN vs. UKBS	-10.56	3.49	-17.4	-3.72	2.47E-03
IVW estimate for potassium~ DBP UKBN vs. UKBS	-2.35	1.34	-4.98	0.29	0.08

Sensitivity analysis using UKB vs. BP consortium including all SNPs before removal of outliers					
Method	Estimate	Standard Error	95% CI		P-value
IVW estimate for Sodium ~ SBP	2.93	1.6	-0.2	6.07	0.07
IVW estimate for Sodium ~ DBP	1.64	0.86	-0.05	3.34	0.06
IVW estimate for Potassium ~SBP	-4.8	3.65	-11.95	2.34	0.19
IVW estimate for Potassium ~ DBP	-1.4	2.02	-5.37	2.57	0.49
