# Supplementary material for: GWAS for urinary sodium and potassium excretion highlights pathways shared with cardiovascular traits
Source: Nat Commun. 2019 Aug 13;10:3653. doi: 10.1038/s41467-019-11451-y (PMC6692500; doi:10.1038/s41467-019-11451-y)
Supplement: Supplementary file 30 — Description of Additional Supplementary Files [file 41467_2019_11451_MOESM30_ESM.pdf]

Supplementary Data Legends

Title: Supplementary Data 1
Description: Genome-wide association results for urinary sodium excretion at $P<1\times 10^{-8}$.

Title: Supplementary Data 2
Description: Genome-wide association results for urinary potassium excretion at $P< 1 \times 10^{-8}$.

Title: Supplementary Data 3
Description: Urinary sodium lead SNPs and corresponding regional SNPs at $P<1\times10^{-8}$.

Title: Supplementary Data 4
Description: Urinary potassium lead SNPs and corresponding regional SNPs at $P<1\times10^{-8}$.

Title: Supplementary Data 5
Description: Urinary sodium SNPs associations and functional annotations at $P<1\times10^{-8}$.

Title: Supplementary Data 6
Description: Urinary potassium SNPs associations and functional annotations at $P<1\times10^{-8}$.

Title: Supplementary Data 7
Description: Association results for urinary sodium excretion lead SNPs after exclusion of medication use and renal diseases (N=262,531).

Title: Supplementary Data 8
Description: Association results for urinary potassium excretion lead SNPs after exclusion of medication use and renal diseases (N=262,531).

Title: Supplementary Data 9
Description: Association of urinary sodium loci with other traits in GWAS Catalog ($P < 5 \times 10^{-8}$).

Title: Supplementary Data 10
Description: Association of urinary potassium loci with other traits in GWAS Catalog ($P < 5 \times 10^{-8}$).

Title: Supplementary Data 11
Description: Phenotypes associated with urinary sodium loci within UK Biobank using GeneAtlas at $P<1\times 10^{-6}$.

Title: Supplementary Data 12
Description: Phenotypes associated with urinary potassium loci within UK Biobank at $P<1\times 10^{-6}$.

Title: Supplementary Data 13
Description: LD score regression between urinary sodium and other traits.

Title: Supplementary Data 14
Description: LD score regression between urinary potassium and other traits.

Title: Supplementary Data 15
Description: Association of urinary sodium loci with SBP and DBP within the UK Biobank study at $p<5\times10^{-8}$.

Title: Supplementary Data 16
Description: Association of urinary potassium loci with SBP and DBP within the UK Biobank study at $P<5\times10^{-8}$.

Title: Supplementary Data 17
Description: Association of urinary sodium and potassium loci with metabolites in phenoscanner ($P < 5 \times 10^{-8}$).

Title: Supplementary Data 18
Description: Association of urinary sodium and potassium loci with metabolites in Airwave using metabolon.

Title: Supplementary Data 19
Description: Summary of eQTL effects of urinary sodium SNPs ($P<1\times10^{-8}$) from eQTL look up using GTeX.

Title: Supplementary Data 20
Description: Summary of eQTL effects of urinary potassium SNPs ($P<1\times10^{-8}$) from eQTL look up using GTeX.

Title: Supplementary Data 21
Description: Ingenuity Pathway Analysis for genen near or eQTL with urinary sodium and potassium excretion.

Title: Supplementary Data 22
Description: Outlier test and effect estimates of urinary sodium loci used for Mendelian randomization analysis.

Title: Supplementary Data 23
Description: Outlier test and effect estimates of urinary potassium loci used for Mendelian randomization analysis.

Title: Supplementary Data 24
Description: Mendelian Randomization analysis for the potential causal effect of urinary sodium on BP and CVD using UK Biobank north vs. south & midland (at P<0.008).

Title: Supplementary Data 25
Description: Mendelian Randomization analysis for the potential causal effect of urinary potassium on BP and CVD using UK Biobank north vs. south & midland (at P<0.008).

Title: Supplementary Data 26
Description: Mendelian Randomization analysis for the potential causal effect of urinary sodium on BP using UK Biobank north vs. south & midland and on CVD using CARDIOGRAM. Focusing on loci involved in significant biologic functions.

Title: Supplementary Data 27
Description: Summary of association results for urinary sodium SNPs among African and Asian ancestries ($P<9.8\times10^{-4}$).
